# The Role of Ultrasonography and Magnetic Resonance Imaging in the Diagnosis of the Adherent Placenta: An Observational Study

**DOI:** 10.7759/cureus.53856

**Published:** 2024-02-08

**Authors:** Rahul Sawant, Swastika Patil, Sanket S Warghade, Siddhant Y Shirsat

**Affiliations:** 1 Radiodiagnosis, Grant Government Medical College and Sir Jamshedjee Jeejeebhoy (JJ) Hospital, Mumbai, IND; 2 Pathology, Smt. Kashibai Navale Medical College and General Hospital, Pune, IND; 3 Radiology, Grant Government Medical College and Sir Jamshedjee Jeejeebhoy (JJ) Hospital, Mumbai, IND

**Keywords:** adherent placenta, diagnosis, ultrasonography, placenta accreta, mri

## Abstract

Introduction

Placenta accreta is an important factor responsible for maternal morbidity and mortality and is commonly associated with emergent postpartum hysterectomy. The precise prenatal diagnosis of affected pregnancies allows optimal obstetric management. Ultrasonography (USG) and magnetic resonance imaging (MRI) are the only diagnostic modalities available for the prenatal diagnosis of placenta accreta.

Objective

This study aims to evaluate the accuracy of USG and MRI in diagnosing adherent placenta.

Methods

Thirty females with placenta previa or a history of previous cesarean sections were evaluated with USG at 28-30 weeks, followed by MRI. The findings of USG and MRI were compared with the intra-operative findings (gold standard) as determined at surgery and by pathological examination.

Results

Abnormal bridging vessel (n = 24; 80%) was the most common finding seen on USG, whereas abnormal bulge (n = 22; 73.3%) and heterogenous placenta (n = 21; 70%) were the most common findings seen on MRI. The sensitivity of USG and MRI was in the range of 86.7%-92.9% and 92.9%-100%, respectively, in diagnosing three types of adherent placenta. The positive predictive values (PPV) of USG and MRI were in the range of 86.7%-86.7% and 93.8%-100%, respectively, in diagnosing three types of adherent placenta. The accuracy of USG and MRI was in the range of 86.7%-96.7% and 96.7%-100%, respectively, in diagnosing three types of adherent placenta.

Conclusion

MRI helps to accurately classify placental invasion according to depth, as can be seen from the results of the present study, where the MRI technique was more accurate in diagnosing three types of adherent placenta.

## Introduction

The adherent placenta encompasses the entire placenta accreta spectrum (PAS). Placenta accreta is the abnormal attachment of the placenta to the uterine wall. It is a condition where the placenta grows deeply into the uterine wall during pregnancy. Placenta accreta is the most common reason for urgent postpartum hysterectomy, which causes considerable maternal morbidity and mortality [1].

Placenta accreta is an aberrant attachment of the placenta to the myometrium caused by chorionic villi invading the myometrium due to a decidua basalis deficit. The depth of myometrial invasion divides placenta accreta into three types called "placenta accreta spectrum (PAS)." In placenta accreta vera, villi are attached to the myometrium, but no muscle invasion exists. Villi in the placenta increta penetrate partly into the myometrium. Placenta percreta is the least common of the three, in which villi penetrate the entire thickness of the myometrium or even beyond the serosa [2].

The etiology of placenta accreta is unknown. Several theories have been proposed for aberrant placentation, including incorrect or excessive trophoblast invasion and localized oxygen tension anomalies [3,4]. Another explanation is that due to a lack of decidualization, the normal physiologic conversion of maternal arteries is lacking [5].

Prior lower-segment cesarean section (LSCS) and placenta previa are the two most common risk factors for placenta accreta. Maternal age, uterine anomalies, previous uterine surgery, dilatation and curettage, and myomectomy are all additional but minor risk factors. In their second trimester, females with placental adhesion showed abnormally high levels of fetoprotein and human chorionic gonadotropin. In the presence of aberrant placentation in any form, these biological indications are frequently elevated [6-9].

Ultrasonography (USG) and magnetic resonance imaging (MRI) are the safest diagnostic modalities for placenta accreta [10,11]. The accurate prenatal identification of placenta accreta allows for optimal management as the timing of delivery, availability of blood products, and recruitment of skilled anesthesia and surgical teams can be arranged in advance [12-14]. Therefore, this was planned to evaluate the role of ultrasonography and MRI in the early diagnosis of placenta accreta. The objectives were to describe the signs of abnormal placentation via USG and MRI and compare the diagnostic accuracy of USG and MRI in the detection of adherent placenta.

## Materials and methods

Study design, study setting, and study duration

The present cross-sectional study was carried out at a tertiary care hospital in a metropolitan city for 12 months.

Study population

On average, 30 cases of placenta accreta were diagnosed in our tertiary care hospital during the last 12 months. So, by the complete enumeration method, the sample size of the study was 30. High-risk pregnant females having clinical suspicion of placenta accreta who were referred to the Department of Radiodiagnosis were included in the study.

Inclusion criteria

The patients with placenta previa and previous LSCS with evidence of adherent placenta on USG were included.

Exclusion criteria

Patients having cardiac pacemakers, prosthetic heart valves, cochlear implants, or any metallic implants; patients having a history of claustrophobia; patients who did not consent to be part of the study; and the patients with placenta previa and previous cesarean sections with no evidence of adherent placenta on USG were excluded.

Ethical considerations

Before the start of the study, a protocol was submitted to the Institutional Ethics Committee of the Grant Government Medical College, Mumbai, and ethical clearance was obtained (IEC/Pharm/RP/281/Mar/2020). Proper informed consent was taken from the patients after explaining to them the risks and benefits of the examination. After explaining that prenatal sex determination would not be done during the investigation, the Pre-Conception and Pre-Natal Diagnostic Techniques (PCPNDT) "F" form was signed by the patient and one witness.

Study procedure

After taking informed consent and signing the PCPNDT "F" form, high-risk pregnant females were investigated with USG (RS80A Ultrasound Machine, Samsung Healthcare, Seoul, South Korea). Only those pregnant females with placenta previa and previous LSCS who had evidence of adherent placenta on USG were investigated with MRI (Magnetom Aera 1.5T, Siemens Medical Solutions USA, Inc., Malvern, PA). Essential clinical history was obtained, and relevant data was collected at the time when the patient was undergoing a scan. T2-weighted half-Fourier acquisition single-shot turbo spin-echo (HASTE) and true fast imaging with steady-state precession (TrueFISP) sequences and T1-weighted gradient echo sequence were acquired in the axial, sagittal, and coronal planes using Siemens' Magnetom Aera 1.5T scanner. The readers who interpreted the findings of the MRI were blinded to the USG reports and USG images. All the findings were documents. All the females were followed up until the delivery of the baby. The findings were compared with the intra-operative findings (considered as gold standard) of the same patient.

Statistical analysis

Data was entered into Microsoft Excel (Windows 11, version 2016, Microsoft® Corp., Redmond, WA), and analyses were done using the Statistical Package for Social Sciences (SPSS) for Windows software (version 26.0, IBM SPSS Statistics, Armonk, NY). Descriptive statistics such as mean and standard deviation (SD) for continuous variables, frequencies, and percentages were calculated for categorical variables. The association between variables was analyzed using the chi-square test for categorical variables. Sensitivity, specificity, and predictive accuracy were calculated for USG and MRI for the diagnosis of adherent placenta by taking the intra-operative findings as the gold standard. The level of significance was set at 0.05.

## Results

**Figure 1:**
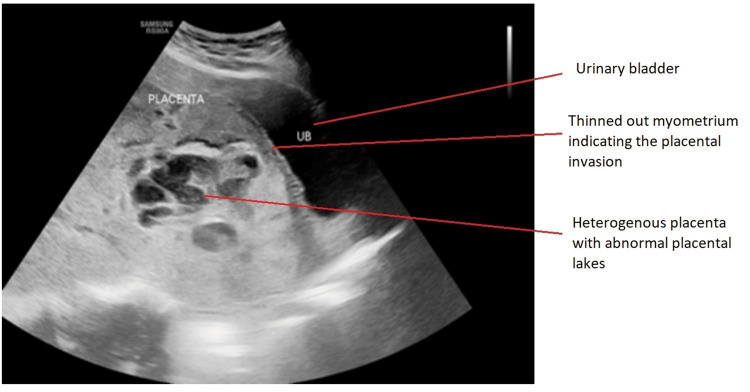
USG scan of the placenta USG: ultrasonography

Figure 1 presents the image of the USG scans of one of the study subjects showing the heterogenous placenta with abnormal placental lakes.

**Figure 2:**
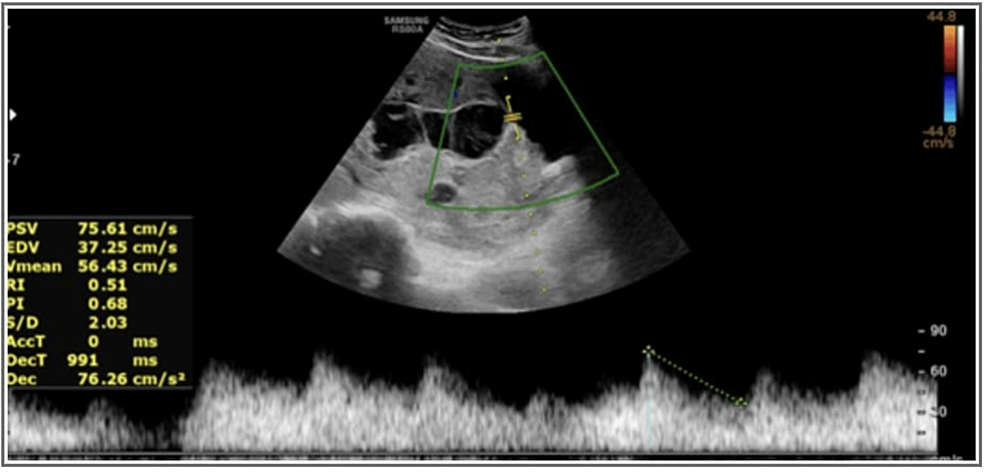
USG Doppler scan image USG: ultrasonography

Figure 2 presents the image of the USG Doppler scan of the same subject suggestive of a vessel traversing the placental-myometrial junction and myometrial-bladder interface, which shows high peak velocity suggestive of the adherent placenta.

**Figure 3:**
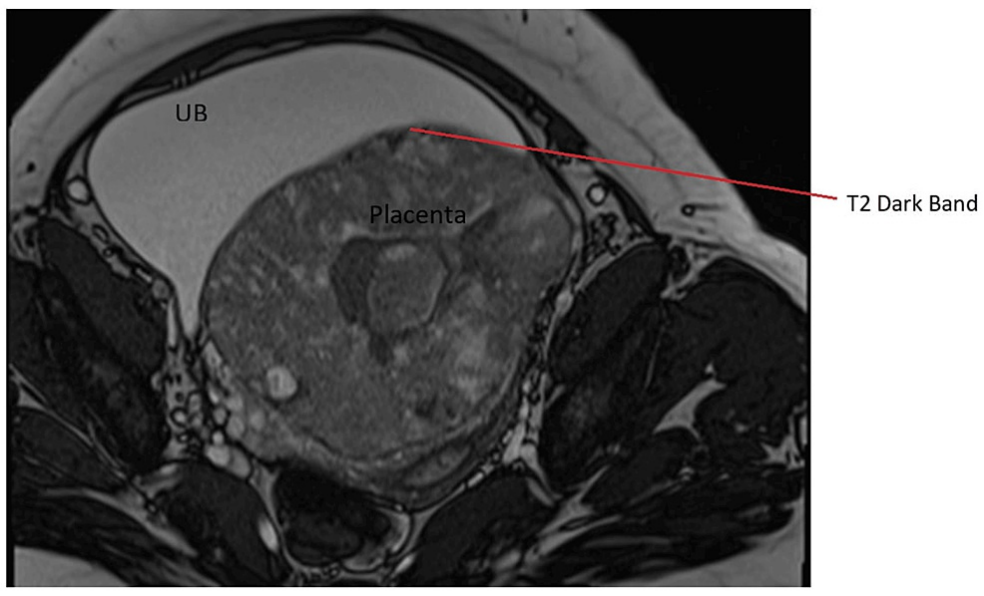
MRI scan image (axial view) Axial view showing a T2 dark band and placental bulging into the posterior wall of the urinary bladder (UB) with a loss of placental-myometrial junction MRI: magnetic resonance imaging

**Figure 4:**
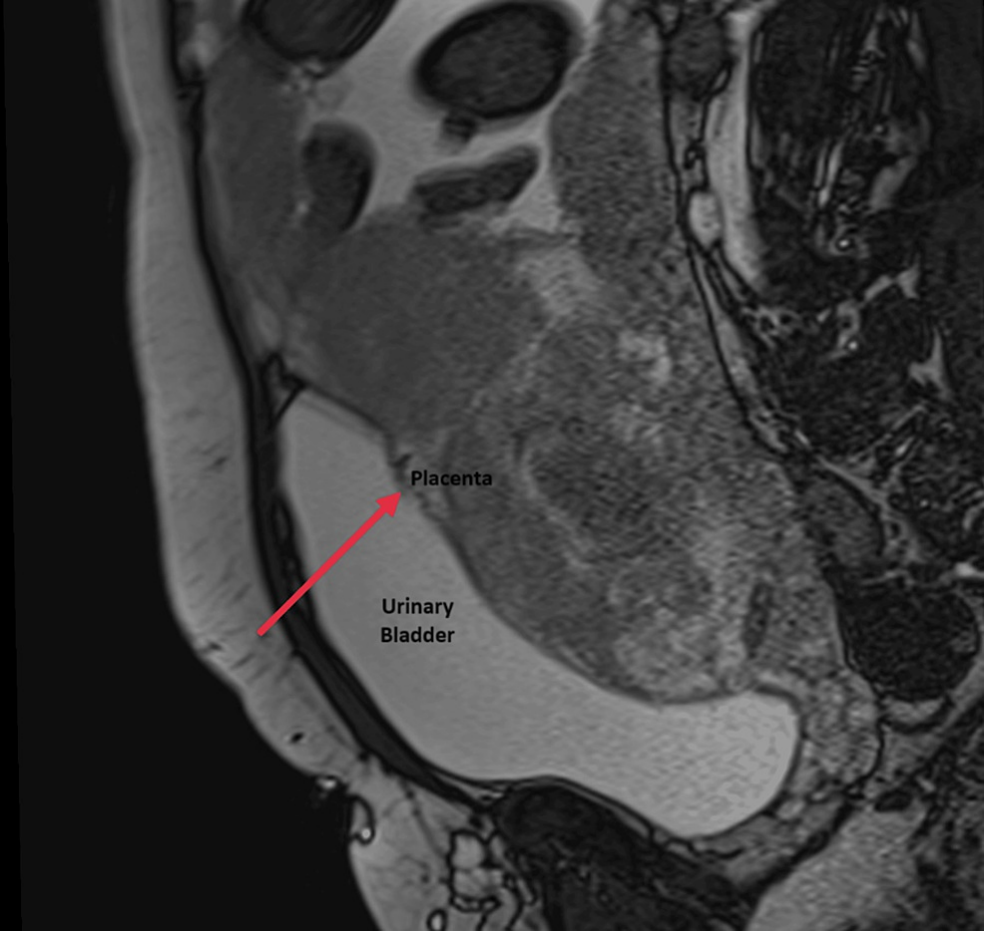
MRI scan image (sagittal view) The arrow showing the heterogenous placenta invading the posterosuperior border of the urinary bladder through the myometrium MRI: magnetic resonance imaging

Figure 3 and Figure 4 present the images of the axial and sagittal views of the MRI scans, respectively.

According to the images, the USG findings showed the thinning of myometrial and abnormal lacunae with turbulent flow suggestive of placenta increta, whereas the MRI findings showed heterogenous placenta and abnormal placental bulge along the posterosuperior wall of the urinary bladder suggestive of placenta percreta. The intra-operative findings were suggestive of placenta percreta.

Table 1 presents the details of the distribution of the study subjects according to age, gestational age, and risk factors. The average age and gestational age of the study participants were 26.67 years and 30.90 years, respectively. All 30 subjects were diagnosed with placenta previa, whereas 29 subjects had a previous history of LSCS.

**Table 1:** Mean age and mean gestational age of the study subjects and number of cases according to the risk factor LSCS, lower-segment cesarean section; SD, standard deviation

Variable	Category	Mean ± SD	Range
Age in years, n (%)	23-25, 8 (26.7%)	26.67 ± 1.80	23-30
	26-30, 22 (73.3%)		
Gestational age in years, n (%)	28-32, 24 (80%)	30.90 ± 2.18	28-35
	33-35, 6 (20%)		
Risk factors, n (%)	Placenta previa, 30 (100%)	-	-
	Previous LSCS, 29 (96.7%)		

Table 2 presents the distribution of the study subjects according to the USG and MRI findings. The USG findings showed that 24 subjects (80%) were diagnosed with abnormal bridging vessels. Myometrial thinning, the loss of retroplacental clear space, and lacunae with turbulent flow were seen in 15 subjects (50%), and bladder wall interruption was seen in six subjects (20%). Abnormal bulge on MRI investigation was seen in 22 subjects (73.3%), heterogeneous placenta was seen in 21 subjects (70%), and T2 hypointense bands were seen in 12 subjects (40%).

**Table 2:** Distribution of the study subjects according to the USG and MRI findings The results have been presented as numbers (n) and percentages (%) USG, ultrasonography; MRI, magnetic resonance imaging

Method	Finding	n	%
USG	Abnormal bridging vessels	24	80.0
	Myometrial thinning	15	50.0
	Loss of retroplacental clear space	15	50.0
	Lacunae with turbulent flow	15	50.0
	Bladder wall interruption	6	20.0
MRI	Abnormal bulge	22	73.3
	Heterogeneous placenta	21	70.0
	T2 hypointense bands	12	40.0

Table 3 presents the details of the distribution of the study subjects according to placenta accreta spectrum. USG examination diagnosed 15 subjects (50%) with placenta increta and 15 subjects (50%) with placenta percreta. MRI findings showed that 16 subjects (53.3%) were diagnosed with placenta increta, 13 subjects (43.3%) with placenta percreta, and one subject (3.4%) with placenta accreta vera. Intra-operative findings showed that 15 subjects (50%) were diagnosed with placenta increta, 13 subjects (43.3%) with placenta percreta, and one subject (3.4%) with placenta accreta vera. Intra-operative findings showed that 15 subjects (50%) were diagnosed with placenta increta, 14 subjects (46.6%) with placenta percreta, and one subject (3.4%) with placenta accreta vera.

**Table 3:** Distribution of the study subjects according to placenta accreta spectrum The results have been presented as numbers (N) and percentages (%) USG, ultrasonography; MRI, magnetic resonance imaging

Type of adherent placenta	USG	MRI	Intra-operative findings
Placenta increta	15 (50%)	16 (53.3%)	15 (50%)
Placenta percreta	15 (50%)	13 (43.3%)	14 (46.6%)
Placenta accreta vera	0 (0%)	1 (3.4%)	1 (3.4%)

Table 4 presents the results of the sensitivity, specificity, and predictive accuracy of USG and MRI in the diagnosis of placenta accreta spectrum. In diagnosing placenta increta, the MRI technique showed significantly greater sensitivity (p < 0.001), negative predictive value (NPV) (p < 0.001), and accuracy (p = 0.009) as compared to the USG method. In diagnosing placenta percreta, the MRI technique showed significantly greater specificity (p < 0.001), positive predictive value (PPV) (p < 0.001), and accuracy (p = 0.044) as compared to the USG method. The UGS method failed to diagnose the only case of placenta accreta vera. When compared to USG in diagnosing placenta accreta vera, the MRI technique showed significantly greater sensitivity (p < 0.001).

**Table 4:** Comparison of the sensitivity, specificity, and predictive accuracy of USG and MRI in the diagnosis of placenta accreta spectrum using the chi-square test *P ≤ 0.05 has been considered as significant USG, ultrasonography; MRI, magnetic resonance imaging; PPV, positive predictive value; NPV, negative predictive value

Test	Increta			Percreta			Accreta vera		
	USG	MRI	p-value	USG	MRI	p-value	USG	MRI	p-value
Sensitivity	86.7%	100.0%	<0.001*	92.9%	92.9%	1.000	0%	100.0%	<0.001*
Specificity	86.7%	93.3%	0.157	87.5%	100.0%	<0.001*	100.0%	100.0%	1.000
PPV	86.7%	93.8%	0.091	86.7%	100.0%	<0.001*	-	100.0%	-
NPV	86.7%	100.0%	<0.001*	93.3%	94.1%	0.774	96.7%	100.0%	0.081
Accuracy	86.7%	96.7%	0.009*	90.0%	96.7%	0.044*	96.7%	100.0%	0.081

Table 5 presents the results of the sensitivity, specificity, and predictive accuracy of USG and MRI in the diagnosis of adherent placenta. The MRI technique showed greater sensitivity (96.7% versus 86.7%), positive predictive value (96.7% versus 86.7%), and accuracy (93.6% versus 76.5%) in diagnosing adherent placenta when compared to the USG method.

**Table 5:** Sensitivity, specificity, and predictive accuracy of USG and MRI in the diagnosis of adherent placenta presented in percentage USG, ultrasonography; MRI, magnetic resonance imaging; PPV, positive predictive value; NPV, negative predictive value

Test	USG	MRI
Sensitivity	86.7%	96.7%
Specificity	0%	0%
PPV	86.7%	96.7%
NPV	0%	0%
Accuracy	76.5%	93.6%

## Discussion

Placenta previa and previous LSCS are the most common risk factors for adherent placenta. Ultrasonography and MRI are the safest modalities used in the diagnosis of the adherent placenta. In the present study, 30 patients were included. All the patients in the study underwent obstetric hysterectomy, and the findings of USG and MRI were compared with intra-operative findings. The results of the study showed that the MRI technique was more reliable and accurate in the diagnosis of the adherent placenta as compared to the USG method.

In the study conducted by Lopes et al. to compare the diagnostic accuracy of USG and MRI in diagnosing the placenta accreta among females with placenta previa, MRI showed greater sensitivity (92.9% versus 87.5%) and PPV (76.5% versus 65.1%) and almost similar specificity (42.9% versus 44.4%) when compared to USG in the diagnosis of placenta accreta [15]. A study conducted by El Wakeel et al. showed that the sensitivity, specificity, and accuracy of MRI in the diagnosis of placenta accreta were 72.73%, 100%, and 86.96%, respectively, whereas the sensitivity, specificity, and accuracy of USG were 63.64%, 91.67%, and 78.26%, respectively [16]. In a study conducted by Davutoğlu et al., the sensitivity, specificity, and diagnostic accuracy of USG and MRI were calculated to be 84.6%, 81.2%, and 82.7% and 100%, 76.9%, and 86.2%, respectively [17]. A study conducted by Hashem et al. to determine the role of MRI and USG in the diagnosis of placental abnormalities showed that MRI had greater sensitivity (80%) and specificity (85.71%) as compared to USG (sensitivity, 68%; specificity, 78.57%) in diagnosing the adherent placenta [18]. In a study conducted by Ashraf et al., MRI showed higher sensitivity, specificity, and PPV as compared to USG in the diagnosis of the low-lying adherent placenta [19]. The authors attributed the high diagnostic accuracy of MRI in the evaluation of the placenta to its exclusive features such as the multiplanar abilities, the range of pulse sequences and parameters that can be used, the better tissue contrast that can be acquired, and the absence of ionizing radiation.

However, a study conducted by Riteau et al. showed that USG had 100% sensitivity in the diagnosis of placenta accreta whereas MRI showed 76.9% sensitivity, and there was a significant difference between both methods (p = 0.03). However, the specificity was 37.5% with USG and 50% for MRI (p  =  0.60) [20]. Similarly, in a study conducted by Dwyer et al., the results showed that the sensitivity, specificity, PPV, and NPV of USG were high as compared to MRI in the diagnosis of placenta accreta [21]. A systematic review and meta-analysis published in 2022 to compare the accuracy of USG and MRI in the diagnosis of placenta accreta spectrum showed that pooled sensitivity and specificity values were similar between USG and MRI. The meta-regression analysis revealed no significant difference in sensitivity (Z = -0.436; p = 0.663) or specificity (Z = 0.055; p = 0.956) between USG and MRI [22]. The authors concluded that MRI is only a complementary diagnostic tool to USG and should be performed when the results of USG are indecisive or inconclusive.

Limitations

The possibility of Berkson's bias cannot be ruled out since the study was conducted in a hospital, and the results found in the current study may differ from the incidence of adherent placenta in the general population.

## Conclusions

The incidence of the adherent placenta has been on the rise because of the increase in the number of cesarian deliveries and placental interventions. USG remains the choice of imaging modality due to its accessibility; however, MRI plays an important role in the diagnosis of placental invasion where USG findings are unclear or unambiguous. MRI helps to accurately classify placental invasion according to the depth that can be seen from the results of the present study where the MRI technique was more accurate in diagnosing the three types of adherent placenta, i.e., accrete vera, increta, and percreta, as compared to USG. This can aid in deciding the treatment and thus will help to reduce the associated morbidity and mortality.
